# Cardiac Magnetic Resonance Imaging in Immune Check-Point Inhibitor Myocarditis: A Systematic Review

**DOI:** 10.3390/jimaging8040099

**Published:** 2022-04-05

**Authors:** Luca Arcari, Giacomo Tini, Giovanni Camastra, Federica Ciolina, Domenico De Santis, Domitilla Russo, Damiano Caruso, Massimiliano Danti, Luca Cacciotti

**Affiliations:** 1 Cardiology Unit, Madre Giuseppina Vannini Hospital, 00177 Rome, Italy; gcamastra@virgilio.it (G.C.); lcuccc@libero.it (L.C.); 2 Cardiology, Clinical and Molecular Medicine Department, Faculty of Medicine and Psychology, Sapienza-University of Rome, 00100 Rome, Italy; giacomo.comotini@gmail.com (G.T.); russo.domitilla@gmail.com (D.R.); 3 Radiology Unit, Madre Giuseppina Vannini Hospital, 00177 Rome, Italy; federica.ciolina@gmail.com (F.C.); massimilianodanti@hotmail.com (M.D.); 4 Radiology Unit, Department of Medical Surgical Sciences and Translational Medicine, Sant’Andrea University Hospital, Sapienza-University of Rome, 00100 Rome, Italy; domenico.desantis@hotmail.it (D.D.S.); damiano.caruso@uniroma1.it (D.C.)

**Keywords:** immune check-point inhibitor, myocarditis, cardiac magnetic resonance imaging, edema, fibrosis, T1 mapping, T2 mapping

## Abstract

Immune checkpoint inhibitors (ICIs) are a family of anticancer drugs in which the immune response elicited against the tumor may involve other organs, including the heart. Cardiac magnetic resonance (CMR) imaging is increasingly used in the diagnostic work-up of myocardial inflammation; recently, several studies investigated the use of CMR in patients with ICI-myocarditis (ICI-M). The aim of the present systematic review is to summarize the available evidence on CMR findings in ICI-M. We searched electronic databases for relevant publications; after screening, six studies were selected, including 166 patients from five cohorts, and further 86 patients from a sub-analysis that were targeted for a tissue mapping assessment. CMR revealed mostly preserved left ventricular ejection fraction; edema prevalence ranged from 9% to 60%; late gadolinium enhancement (LGE) prevalence ranged from 23% to 83%. T1 and T2 mapping assessment were performed in 108 and 104 patients, respectively. When available, the comparison of CMR with endomyocardial biopsy revealed partial agreement between techniques and was higher for native T1 mapping amongst imaging biomarkers. The prognostic assessment was inconsistently assessed; CMR variables independently associated with the outcome included decreasing LVEF and increasing native T1. In conclusion, CMR findings in ICI-M include myocardial dysfunction, edema and fibrosis, though less evident than in more classic forms of myocarditis; native T1 mapping retained the higher concordance with EMB and significant prognostic value.

## 1. Introduction

Cancer immunotherapy has become a well-established and extremely effective treatment option for several cancers [1,2]. This is based on immune checkpoint inhibitors (ICIs), which is a family of drugs that targets the ‘immune checkpoint’, which is a complex of membrane receptors that regulate the immune response. Indeed, while a number of stimulatory and inhibitory ligands and receptors physiologically balance and regulate immune mechanisms, tumors can exploit these pathways to induce immune tolerance to themselves. In this latter setting, ICIs re-modulate the immune checkpoint and awaken an immune response directed against cancer cells [3]. However, the immune response elicited by ICIs is not completely tumor-specific and may enhance the development of immune-related adverse events, involving a number of organs, among which is the heart [4]. Despite being rare, the cardiac immune-related adverse event received vast attention [5,6]. In particular, myocarditis appeared as the main and most important cardiac immune-related adverse event due to ICIs. Reaching a definite diagnosis of ICI-related myocarditis (ICI-M) is crucial, as for such an adverse event ICI therapy must be stopped—which, in turn, means that a wrong diagnosis may lead to inappropriate discontinuation of a life-saving therapy [4]. Cardiac imaging testing offers valid options to guide differential diagnosis between myocarditis, myocardial infarction and other conditions with a comparable clinical presentation. Cardiac magnetic resonance imaging (CMR) is a guideline-recommended examination in patients with myocardial infarction with a non-obstructive coronary artery to provide a non-invasive assessment of differential diagnoses, including takotsubo syndrome and myocarditis [7]. CMR, especially when tissue mapping techniques are available, has a central role in the evaluation of inflammatory heart conditions [8], providing diagnostic as well as prognostic information [9,10]. Very recently, several articles reported on CMR in patients with ICI-M, which aided the diagnostic work-up as well as risk-stratification. The aim of the present study is to provide a systematic review of the existing evidence of CMR in ICI-M.

## 2. Materials and Methods

**Figure 1:**
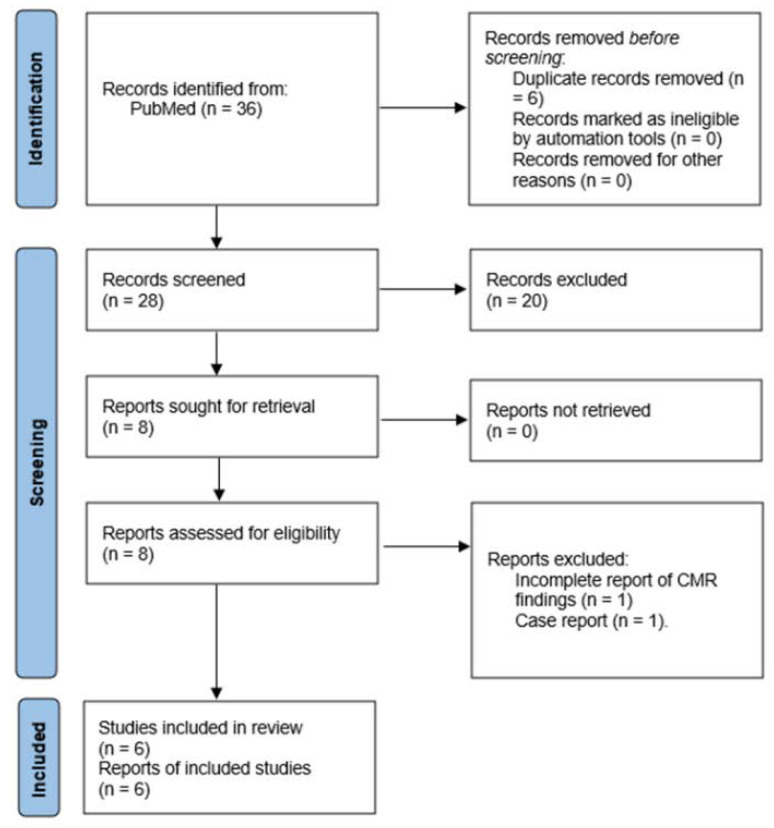
Prisma algorithm depicting the selection process of reviewed studies.

We performed the systematic research on Embase/PubMed using the following strings: [“immune checkpoint inhibitors” AND “myocarditis” AND “magnetic resonance imaging”] and [“immune checkpoint inhibitors” AND cardiotoxicities AND “magnetic resonance imaging”]. The search is updated to 31 January 2021, and retrieved 36 results that were subsequently assessed for inclusion using the PRISMA methodology [11] (Figure 1). As per prespecified criteria, case reports were excluded from the analysis. After screening for eligibility, 6 studies were finally selected [12,13,14,15,16,17]. Two of these [15,16] enrolled patients within a same multicenter registry, albeit providing partly different information (e.g., T1 and T2 mapping in one); for this reason, they were both included in the analysis.

## 3. Cardiac Magnetic Resonance Findings

A summary of the main CMR findings in the analyzed study is reported within Table 1. Overall, 166 patients from 5 cohorts, plus a further 86 patients from a sub-analysis targeted on tissue mapping assessment, were considered. Briefly, CMR revealed a mostly preserved left ventricular ejection fraction. Edema prevalence ranged from 9% to 60% and late gadolinium enhancement (LGE) prevalence ranged from 23% to 83%. The T1 and T2 mapping assessment were performed in 108 and 104 patients, respectively.

### 3.1. Left Ventricular Function

The clinical presentation of ICI-M varied between studies, from subtle myocardial involvement to extensive left ventricular (LV) dysfunction. However, except for the first report from Escudier et al. [12], LV ejection fraction (LVEF) was on average preserved or only mildly impaired. This might be explained by increasing awareness of the syndrome, more careful monitoring and therefore increased diagnostic yield, which led to increased identification of milder forms over time. Patterns of LV dysfunction were inconsistently reported amongst different studies. However, the presence of a takotsubo-like myocardial dysfunction (significant reduction of LVEF with prompt recovery at follow-up) has been reported in a non-trivial number of patients [12]. CMR presentation of these cases might overlap that of the classic takotsubo syndrome [18]; hence, a comprehensive diagnostic work-up, possibly, including endomyocardial biopsy might be necessary to reach a correct diagnosis [19].

### 3.2. Edema

The presence of edema is the hallmark of acute myocardial inflammation, and can be effectively identified and measured non-invasively by means of CMR [8,20]. Rates of edema detection in ICI-M ranged from 27% to 60% in the analyzed study (Table 1). From a technical point of view, several techniques can be used to image myocardial edema. The classic T2-STIR sequence allows a qualitative or semi-quantitative evaluation of myocardial water content, and it has been extensively used in the past years. However, it is affected by several limitations including low signal to noise ratio, not infrequent acquisition of uninterpretable images, and low sensitivity when compared to more recent techniques [21,22]. Recently developed T1 and T2 mapping techniques can provide a more accurate and quantitative assessment of myocardial edema. T2 mapping is a specific marker of increased myocardial water content, whereas T1 mapping (native T1 as well as extra-cellular volume quantification by post-contrast T1 mapping) is a highly sensitive marker of diseased myocardium, albeit less specific, as its increase can be driven by edema as well as fibrosis [23,24]. The rate of edema detection in the analyzed studies was remarkably lower than what was reported in large cohort studies of non-ICI-M myocarditis [25,26], especially when considering that recent and more sensitive mapping sequences were used in two out of the six reviewed papers. This finding might be explained by the greater awareness of ICI-M in recent times, with subsequent implementation of screening strategies leading to increased diagnostic yield amongst milder forms. Moreover, since timing for CMR in ICI-M suspected cases is not defined, it should be taken into consideration that in some instances CMR was performed after the initiation of steroid treatment, having the effect of reducing myocardial inflammation and thus edema [16]. Indeed, in patients with sarcoidosis, Puntmann et al. observed a significant reduction of native T1 and T2, indicating resolving myocardial edema in the patients who underwent steroid treatment [27]. Amongst the analyzed study on ICI-M, one provided systematic data regarding T2 mapping in a large subset of patients [16], which allowed for more accurate detection of underlying myocardial edema. Furthermore, Faron et al. performed CMR scans at baseline and follow-up after a median of 3 months after ICI therapy, finding significant increase in native T1 and T2 mapping despite unchanged [17] results from traditional T2-STIR sequences.

Overall, the available published data indicate that, to a certain extent differently from the classic myocarditis phenotype, myocardial edema in ICI-M is often subtle if not totally absent, especially in the context of ongoing steroid therapy. Hence, T1 and T2 mapping techniques might be needed to provide a more reliable CMR diagnostic evaluation in this setting.

### 3.3. Fibrosis

**Figure 2:**
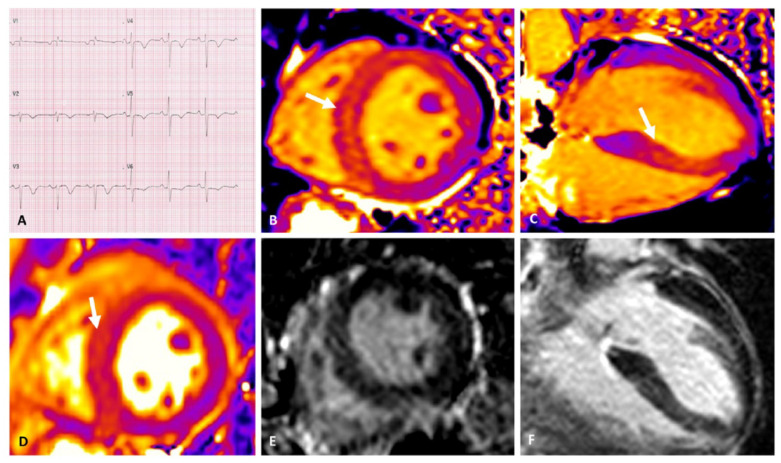
ECG with diffuse T- wave inversion (**A**). Native T1 mapping, higher signal detected within the interventricular septum in the mid-short axis (arrow in (**B**)) and in 4-chamber view (arrow in (**C**)); in the same area; T2 mapping shows higher signal intensity indicating edema (arrow in (**D**)). Absence of replacement fibrosis as assessed by LGE (**E**,**F**). Adapted with permission from [18].

Late gadolinium enhancement (LGE) is a technique in which CMR images are acquired minutes after the injection of the contrast media, so that appropriate washout from the healthy tissue has taken place and bright areas with increased gadolinium concentration appear brighter, grossly representing the amount of irreversible injury or scar [28]. In myocarditis, non-ischemic type of LGE is highly prevalent, and reduces to a certain amount over time because of overestimation due to myocardial edema in the acute phase [29]. As with edema, in ICI-M the observed prevalence of LGE (Table 1) is lower than expected from large studies on myocarditis [25,26], and therefore might be absent (Figure 2). Zhang et al. discussed this finding, observing how lower LGE detection rates were linked to CMR examinations performed early in the clinical course, possibly indicating that at least a few days could be needed to reach the final amount of cardiac involvement determined by myocarditis [15]. In the same study, neither T2-STIR findings, LGE nor EMB results, discriminated the outcome, whereas LVEF remained the only variable independently associated with major adverse cardiac events at the follow-up. Native T1 is a very sensitive imaging biomarkers that has been linked to diffuse myocardial fibrosis, with histology validation available in several diseases [30,31]; on the other hand, it has a rather low specificity, where its increase can also be driven by the presence of myocardial edema [32,33]. In patients receiving ICI treatment, tissue mapping by CMR was able to detect myocardial changes that were unnoticed by using standard LGE imaging [17]. Amongst the analyzed study on ICI-M, only one provided prognostic data regarding T1 mapping [16]. The authors found that native T1 values remained independently associated to the outcome; moreover, though found in a minority of the patients within the cohort, normal native T1 represented a good prognostic sign, with no major cardiovascular events taking place in this subset of patients. Additional results coming from this same study demonstrated a correlation between T1 and T2 values in this cohort [34]. Hence, it cannot be excluded that, in this case, the native T1 increase is linked to remnant myocardial edema rather than to fibrosis [35].

## 4. Clinical Implications

Awareness toward ICI-related myocarditis increased exponentially after the first descriptions of this potentially fatal adverse event. These observations prompted the incorporation of algorithms for ICI-related myocarditis’ recognition and treatment by oncologic societies [36,37]. These algorithms were centered on the active screening of myocarditis with troponin blood testing and to promptly withhold ICI treatment in the case of suspected or certain myocarditis diagnosis. Such a pro-active screening strategy was motivated by the fulminant, life-threatening nature of the first ICI-related myocarditis reports [3,38,39,40]. Moreover, in general, myocarditis can be difficult to diagnose, as it generally presents with non-specific signs and symptoms, and, also, may rapidly complicate with acute heart failure and arrhythmias [41]. 

However, a troponin-based myocarditis screening has inherent limitations [42]. Troponins may raise due to a number of different cardiac (but also non-cardiac) conditions [43,44,45] and should always be interpreted within the underlying clinical setting. Some ICIs recipients may be at higher risk for (and thus with higher odds of developing) myocardial ischemia rather than myocarditis (i.e., patients with lung cancer)—this further complicates troponin interpretation. Furthermore, additional data have shown that the clinical spectrum of ICI-related myocarditis is wider than initially thought, and sub-clinical, asymptomatic cases may occur [46,47]. Thus, in most recent guidelines, troponin-based screening is no longer recommended [48]. Clinical assessment and close collaboration between oncologists and cardiologists are therefore fundamental in determining a diagnosis of ICI-related myocarditis. When approaching differential diagnosis, some aspects should be considered to help in guiding the clinical evaluation. It has been reported that ICI-related myocarditis occurs more frequently within the first weeks of treatment and usually with combination therapy (i.e., therapeutic scheme with more than one ICIs) [4,36,37]. Moreover, myocarditis due to ICIs is commonly accompanied by myositis and/or a myasthenia gravis-like syndrome [48] and the assessment of creatine phosphokinase (CPK) values may help interpreting an abnormal troponin testing [4].

**Figure 3:**
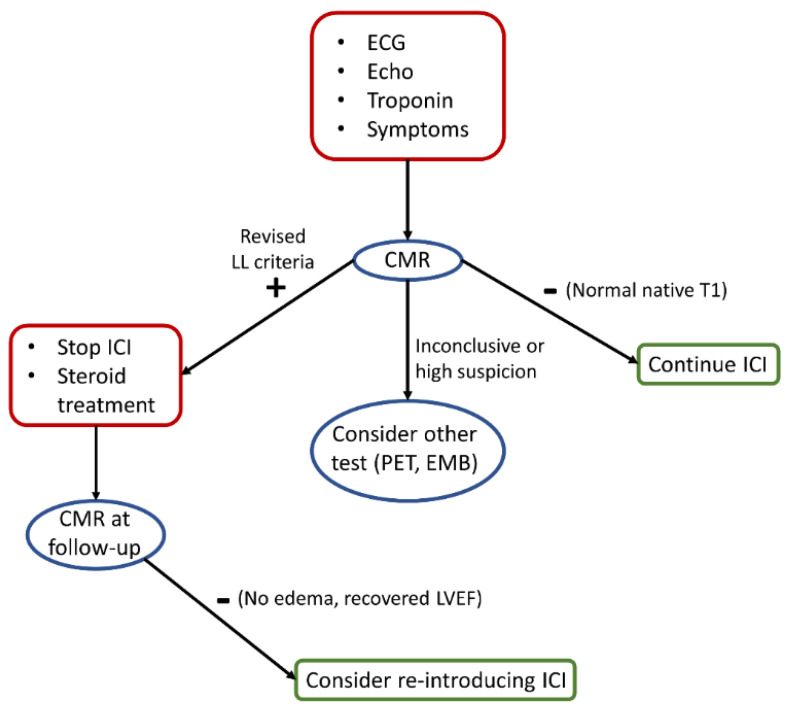
Proposed diagnostic algorithm for the evaluation of patients with suspected ICI-M. We hypothesize a CMR-centered algorithm, with the examination promptly performed according to clinical red flags (red square on top). Results from the CMR examination can further guide the diagnostic work-up, with other second-line tests potentially available in the case of inconclusive findings. The prognostic relevance of CMR biomarkers might be used as gatekeepers for a further re-challenge with ICI.

In this context, CMR examination plays a pivotal role to explore a suspected ICI-M under a clinical suspicion (Figure 3). Symptoms, increased troponin level and newly detected echocardiographic (ECG) abnormalities might suggest the need to perform CMR; additionally, electrocardiographic changes have been described after the baseline in patients with ICI-M, including prolonged QRS and QT interval duration, sinus tachycardia, decreased QRS voltages and repolarization abnormalities [49]. Hence, it might be reasonable to plan an imaging test after detecting new ECG abnormalities as compared to the baseline. CMR offers several tools for the evaluation of non-ischemic myocardial inflammation, whose diagnostic criteria have been recently updated [8]; specifically, the revised Lake Louise (LL) criteria now include the more recent T1 and T2 mapping techniques—this could have relevant implications for ICI-M. As shown by the aforementioned studies, myocardial changes can be often mild and potentially undetectable without using mapping tools. Accordingly, the agreement with EMB was relatively low in the study by Zhang et al., in which 50–60% of the patients with biopsy-proven myocarditis had negative T2-STIR and LGE imaging [15]; however, a further analysis from the same cohort and including only patients with an available tissue mapping evaluation demonstrated that at least one of the main modified LL criteria was present in all of the patients, and more than 80% of patients with biopsy-proven myocarditis had abnormal native T1 [16]. A negative scan, especially in the presence of normal native T1, should prompt reassurance, especially when considering the very low-risk of this subset of patients [16]. On the other hand, where LL criteria should not be met, albeit there remains clinical suspicion with inconclusive CMR examination, further tests such as EMB and positron emission tomography could be considered [50,51]. In the presence of a CMR-confirmed ICI-M diagnosis, the drug should be halted and treatment with steroids commenced. A CMR at follow-up can be considered to clarify the resolution of the disease, signified by regression of myocardial edema [52], or conversely, the evolution towards remodeling [53]. In patients undergoing cancer-related treatment, Haslbauer et al. observed a parallel increase in native T1 and T2 values when CMR scans were performed early after treatment initiation [54], indicating underlying myocardial edema; on the other hand, cases that underwent a late CMR examination (more than 12 months post-treatment) were more likely to have increased native T1 and lower T2 values, indicating fibrosis and cardiac remodeling. Re-challenge with ICI is currently a debated topic, and it has been hypothesized that reintroduction in low-grade patients might be a feasible option, albeit better definition of the grading is needed [55]. Potentially, CMR imaging biomarkers, especially the prognostic relevant native T1, could serve as criteria for considering a patient eligible for re-challenge with ICI.

## 5. Limitations

The present constitutes the first systematic review on a topic in which still few data are currently available, with a relatively heterogenous population and CMR protocols used amongst different studies; this makes our considerations exploratory and in need of further data to be confirmed. Accordingly, larger sample sizes and more homogeneity amongst studies are needed to make a meta-analysis approach potentially reliable.

## 6. Conclusions

CMR findings in ICI-M include myocardial dysfunction, edema and fibrosis, albeit less pronounced than in more classic forms of myocarditis. A comprehensive CMR examination, including T1 and T2 mapping, can provide additional information, with a prognostic value being potentially useful in guiding clinical management.

## Figures and Tables

**Table 1:** CMR characteristics of patients enrolled in the selected studies.

Study (First Author)	Patients	LVEF (%)	Edema (%)	T1 and T2 Mapping	LGE (%)	Prognostic Assessment
Escudier et al.	n = 15	35 (15, 73)	5/15 (33)	No	3/13 (23)	No
Guo et al.	n = 6	55 (31, 60)	3/6 (50)	No	5/6 (83)	No
Higgins et al.	n = 20	53 (39, 60)	12/20 (60)	T2 in n = 3	14 (70)	No
Zhang et al.	n = 103	49 ± 15	28/103 (27)	No	49/103 (48)	Yes
Thavendiranathan et al.	n = 86	51 ± 14	42/79 (34)	T1 (n = 86), T2 (n = 79)	48/86 (56)	Yes
Faron et al.	n = 22	59 ± 14	2 (9)	Yes	9 (41)	No

## References

[1] Wolchok J.D. (2015). Bench to Bedside PD-1 Blockers. Cell.

[2] Postow M.A., Callahan M.K., Wolchok J.D. (2015). Immune Checkpoint Blockade in Cancer Therapy. J. Clin. Oncol..

[3] Spallarossa P., Meliota G., Brunelli C., Arboscello E., Ameri P., Dessalvi C.C., Grossi F., Deidda M., Mele D., Sarocchi M. (2018). Potential cardiac risk of immune-checkpoint blockade as anticancer treatment: What we know, what we do not know, and what we can do to prevent adverse effects. Med. Res. Rev..

[4] Spallarossa P., Sarocchi M., Tini G., Arboscello E., Toma M., Ameri P., Porto I. (2020). How to Monitor Cardiac Complications of Immune Checkpoint Inhibitor Therapy. Front. Pharmacol..

[5] Hu J.R., Florido R., Lipson E.J., Naidoo J., Ardehali R., Tocchetti C.G., Lyon A.R., Padera R.F., Johnson D.B., Moslehi J. (2019). Cardiovascular toxicities associated with immune checkpoint inhibitors. Cardiovasc. Res..

[6] Salem J.E., Manouchehri A., Moey M., Lebrun-Vignes B., Bastarache L., Pariente A., Gobert A., Spano J.-P., Balko J.M., Bonaca M.P. (2018). Cardiovascular toxicities associated with immune checkpoint inhibitors: An observational, retrospective, pharmacovigilance study. Lancet Oncol..

[7] Collet J.-P., Thiele H., Barbato E., Barthélémy O., Bauersachs J., Bhatt D.L., Dendale P., Dorobantu M., Edvardsen T., Folliguet T. (2021). 2020 ESC Guidelines for the management of acute coronary syndromes in patients presenting without persistent ST-segment elevation. Eur. Heart J..

[8] Ferreira V.M., Schulz-Menger J., Holmvang G., Kramer C.M., Carbone I., Sechtem U., Kindermann I., Gutberlet M., Cooper L., Liu P. (2018). Cardiovascular Magnetic Resonance in Nonischemic Myocardial Inflammation: Expert Recommendations. J. Am. Coll. Cardiol..

[9] Winau L., Hinojar Baydes R., Braner A., Drott U., Burkhardt H., Sangle S., D’Cruz D.P., Carr-White G., Marber M., Schnoes K. (2018). High-sensitive troponin is associated with subclinical imaging biosignature of inflammatory cardiovascular involvement in systemic lupus erythematosus. Ann. Rheum. Dis..

[10] de Leuw P., Arendt C.T., Haberl A.E., Froadinadl D., Kann G., Wolf T., Stephan C., Schuettfort G., Vasquez M., Arcari L. (2021). Myocardial Fibrosis and Inflammation by CMR Predict Cardiovascular Outcome in People Living With HIV. JACC Cardiovasc. Imaging.

[11] Page M.J., McKenzie J.E., Bossuyt P.M., Boutron I., Hoffmann T.C., Mulrow C.D., Shamseer L., Tetzlaff J.M., Akl E.A., Brennan S.E. (2021). The PRISMA 2020 statement: An updated guideline for reporting systematic reviews. BMJ.

[12] Escudier M., Cautela J., Malissen N., Ancedy Y., Orabona M., Pinto J., Monestier S., Grob J.-J., Scemama U., Jacquier A. (2017). Clinical features, management, and outcomes of immune checkpoint inhibitor-related cardiotoxicity. Circulation.

[13] Guo C.W., Alexander M., Dib Y., Lau P.K.H., Weppler A.M., Au-Yeung G., Lee B., Khoo C., Mooney D., Joshi S.B. (2020). A closer look at immune-mediated myocarditis in the era of combined checkpoint blockade and targeted therapies. Eur. J. Cancer.

[14] Higgins A.Y., Arbune A., Soufer A., Ragheb E., Kwan J.M., Lamy J., Henry M., Cuomo J.R., Charifa A., Gallegos C. (2021). Left ventricular myocardial strain and tissue characterization by cardiac magnetic resonance imaging in immune checkpoint inhibitor associated cardiotoxicity. PLoS ONE.

[15] Zhang L., Awadalla M., Mahmood S.S., Nohria A., Hassan M.Z.O., Thuny F., Zlotoff D.A., Murphy S.P., Stone J.R., Golden D.L.A. (2020). Cardiovascular magnetic resonance in immune checkpoint inhibitor-associated myocarditis. Eur. Heart J..

[16] Thavendiranathan P., Zhang L., Zafar A., Drobni Z.D., Mahmood S.S., Cabral M., Awadalla M., Nohria A., Zlotoff D.A., Thuny F. (2021). Myocardial T1 and T2 Mapping by Magnetic Resonance in Patients With Immune Checkpoint Inhibitor–Associated Myocarditis. J. Am. Coll. Cardiol..

[17] Faron A., Isaak A., Mesropyan N., Reinert M., Schwab K., Sirokay J., Sprinkart A.M., Bauernfeind F.-G., Dabir D., Pieper C.C. (2021). Cardiac mri depicts immune checkpoint inhibitor-induced myocarditis: A prospective study. Radiology.

[18] Camastra G., Arcari L., Ciolina F., Danti M., Cacciotti L. (2020). Cardiac magnetic resonance imaging of transient myocardial dysfunction in a patient treated with checkpoint-targeted immunotherapy. Eur. J. Cancer.

[19] Palaskas N.L., Segura A., Lelenwa L., Siddiqui B.A., Subudhi S.K., Lopez-Mattei J., Durand J.B., Deswal A., Zhao B., Buja L.M. (2021). Immune checkpoint inhibitor myocarditis: Elucidating the spectrum of disease through endomyocardial biopsy. Eur. J. Heart Fail..

[20] Fernández-Jiménez R., Sánchez-González J., Aguero J., Del Trigo M., Galán-Arriola C., Fuster V., Ibáñez B. (2015). Fast T2 gradient-spin-echo (T2-GraSE) mapping for myocardial edema quantification: First in vivo validation in a porcine model of ischemia/reperfusion. J. Cardiovasc. Magn. Reson..

[21] McAlindon E.J., Pufulete M., Harris J.M., Lawton C.B., Moon J.C., Manghat N., Hamilton M.C.K., Weale P., Bucciarelli-Ducci C. (2015). Measurement of Myocardium at Risk with Cardiovascular MR: Comparison of Techniques for Edema Imaging. Radiology.

[22] Arcari L., Bucciarelli-Ducci C., Francone M., Agati L. (2018). Myocardial Salvage Imaging: Where Are We and Where Are We Heading? A Cardiac Magnetic Resonance Perspective. Curr. Cardiovasc. Imaging Rep..

[23] Puntmann V.O., Peker E., Chandrashekhar Y., Nagel E. (2016). T1 Mapping in Characterizing Myocardial Disease. Circ. Res..

[24] Arcari L., Engel J., Freiwald T., Zhou H., Zainal H., Gawor M., Buettner S., Geiger H., Hauser I., Nagel E. (2021). Cardiac biomarkers in chronic kidney disease are independently associated with myocardial edema and diffuse fibrosis by cardiovascular magnetic resonance. J. Cardiovasc. Magn. Reson..

[25] Aquaro G.D., Perfetti M., Camastra G., Monti L., Dellegrottaglie S., Moro C., Pepe A., Todiere G., Lanzillo C., Scatteia A. (2017). Cardiac MR With Late Gadolinium Enhancement in Acute Myocarditis With Preserved Systolic Function: ITAMY Study. J. Am. Coll. Cardiol..

[26] Hinojar R., Foote L., Ucar E.A., Jackson T., Jabbour A., Yu C.Y., McCrohon J., Higgins D., Carr-White G., Mayr M. (2015). Native T1 in discrimination of acute and convalescent stages in patients with clinical diagnosis of myocarditis: A proposed diagnostic algorithm using CMR. JACC Cardiovasc. Imaging.

[27] Puntmann V.O., Isted A., Hinojar R., Foote L., Carr-White G., Nagel E. (2017). T1 and T2 mapping in recognition of early cardiac involvement in systemic sarcoidosis. Radiology.

[28] Kim R.J., Wu E., Rafael A., Chen E.L., Parker M.A., Simonetti O., Klocke F.J., Bonow R.O., Judd R.M. (2000). The use of contrast-enhanced magnetic resonance imaging to identify reversible myocardial dysfunction. N. Engl. J. Med..

[29] Aquaro G.D., Ghebru Habtemicael Y., Camastra G., Monti L., Dellegrottaglie S., Moro C. (2019). Prognostic Value of Repeating Cardiac Magnetic Resonance in Patients With Acute Myocarditis. J. Am. Coll. Cardiol..

[30] Nakamori S., Dohi K., Ishida M., Goto Y., Imanaka-Yoshida K., Omori T., Goto I., Kumagai N., Fujimoto N., Ichikawa Y. (2018). Native T1 Mapping and Extracellular Volume Mapping for the Assessment of Diffuse Myocardial Fibrosis in Dilated Cardiomyopathy. JACC Cardiovasc. Imaging.

[31] Child N., Suna G., Dabir D., Yap M.-L., Rogers T., Kathirgamanathan M., Arroyo-Ucar E., Hinojar R., Mahmoud I., Young C. (2018). Comparison of MOLLI, shMOLLLI, and SASHA in discrimination between health and disease and relationship with histologically derived collagen volume fraction. Eur. Heart J. Cardiovasc. Imaging.

[32] Ferreira V.M., Piechnik S.K., Dall’Armellina E., Karamitsos T.D., Francis J.M., Choudhury R.P., Friedrich M.G., Robson M.D., Neubauer S. (2012). Non-contrast T1-mapping detects acute myocardial edema with high diagnostic accuracy: A comparison to T2-weighted cardiovascular magnetic resonance. J. Cardiovasc. Magn. Reson..

[33] Arcari L., Hinojar R., Engel J., Freiwald T., Platschek S., Zainal H., Zhou H., Vasquez M., Keller T., Rolf A. (2020). Native T1 and T2 provide distinctive signatures in hypertrophic cardiac conditions–Comparison of uremic, hypertensive and hypertrophic cardiomyopathy. Int. J. Cardiol..

[34] Thavendiranathan P., Zhang L., Neilan T.G. (2021). Reply: Imaging Edema in Immune Checkpoint Inhibitor Myocarditis: A Moving Target. J. Am. Coll. Cardiol..

[35] Arcari L., Camastra G., Ciolina F., Danti M., Cacciotti L. (2021). Imaging Edema in Immune Checkpoint Inhibitor Myocarditis: A Moving Target. J. Am. Coll. Cardiol..

[36] Puzanov I., Diab A., Abdallah K., Bingham C.O., Brogdon C., Dadu R., Hamad L., Kim S., Lacouture M.E., LeBoeuf N.R. (2017). Managing toxicities associated with immune checkpoint inhibitors: Consensus recommendations from the Society for Immunotherapy of Cancer (SITC) Toxicity Management Working Group. J. Immunother. Cancer.

[37] Brahmer J.R., Lacchetti C., Schneider B.J., Atkins M.B., Brassil K.J., Caterino J.M., Chau I., Ernstoff M.S., Gardner J.M., Ginex P. (2018). Management of immune-related adverse events in patients treated with immune checkpoint inhibitor therapy: American society of clinical oncology clinical practice guideline. J. Clin. Oncol..

[38] Johnson D.B., Balko J.M., Compton M.L., Chalkias S., Gorham J., Xu Y., Hicks M., Puzanov I., Alexander M.R., Bloomer T.L. (2016). Fulminant Myocarditis with Combination Immune Checkpoint Blockade. N. Engl. J. Med..

[39] Mahmood S.S., Fradley M.G., Cohen J.V., Nohria A., Reynolds K.L., Heinzerling L.M., Sullivan R.J., Damrongwatanasuk R., Chen C.L., Gupta D. (2018). Myocarditis in Patients Treated With Immune Checkpoint Inhibitors. J. Am. Coll. Cardiol..

[40] Moslehi J.J., Salem J.E., Sosman J.A., Lebrun-Vignes B., Johnson D.B. (2018). Increased reporting of fatal immune checkpoint inhibitor-associated myocarditis. Lancet.

[41] PCaforio A.L., Pankuweit S., Arbustini E., Basso C., Gimeno-Blanes J., Felix S.B., Fu M., Heliö T., Heymans S., Jahns R. (2013). Current state of knowledge on aetiology, diagnosis, management, and therapy of myocarditis: A position statement of the European Society of Cardiology Working Group on Myocardial and Pericardial Diseases. Eur. Heart J..

[42] Spallarossa P., Tini G., Sarocchi M., Arboscello E., Grossi F., Queirolo P., Zoppoli G., Ameri P. (2019). Identification and Management of Immune Checkpoint Inhibitor-Related Myocarditis: Use Troponin Wisely. J. Clin. Oncol..

[43] Januzzi J.L., Filippatos G., Nieminen M., Gheorghiade M. (2012). Troponin elevation in patients with heart failure: On behalf of the third Universal Definition of Myocardial Infarction Global Task Force: Heart Failure Section. Eur. Heart J..

[44] Turan A., Cohen B., Rivas E., Liu L., Pu X., Maheshwari K., Farag E., Onal O., Wang J., Ruetzler K. (2021). Association between postoperative haemoglobin and myocardial injury after noncardiac surgery: A retrospective cohort analysis. Br. J. Anaesth..

[45] Arcari L., Luciani M., Cacciotti L., Pucci M., Musumeci M.B., Pietropaolo L., Spuntarelli V., Negro A., Camastra G., Bentivegna E. (2021). Coronavirus disease 2019 in patients with cardiovascular disease: Clinical features and implications on cardiac biomarkers assessment. J. Cardiovasc. Med..

[46] Sarocchi M., Grossi F., Arboscello E., Bellodi A., Genova C., Dal Bello M.G., Rijavec E., Barletta G., Biello F., Ghigliotti G. (2018). Serial Troponin for Early Detection of Nivolumab Cardiotoxicity in Advanced Non-Small Cell Lung Cancer Patients. Oncologist.

[47] Palaskas N., Lopez-Mattei J., Durand J.B., Iliescu C., Deswal A. (2020). Immune Checkpoint Inhibitor Myocarditis: Pathophysiological Characteristics, Diagnosis, and Treatment. J. Am. Heart Assoc..

[48] Schneider B.J., Naidoo J., Santomasso B.D., Lacchetti C., Adkins S., Anadkat M., Atkins M.B., Brassil K.J., Caterino J.M., Chau I. (2021). Management of Immune-Related Adverse Events in Patients Treated With Immune Checkpoint Inhibitor Therapy: ASCO Guideline Update. J. Clin. Oncol..

[49] Power J.R., Alexandre J., Choudhary A., Ozbay B., Hayek S., Asnani A., Tamura Y., Aras M., Cautela J., Thuny F. (2021). Electrocardiographic Manifestations of Immune Checkpoint Inhibitor Myocarditis. Circulation.

[50] Ederhy S., Devos P., Pinna B., Funck-Brentano E., Abbar B., Fenioux C., Cohen A.A., Moslehi J., Bretagne M., Allenbach Y. (2022). 18F-fluorodeoxyglucose positron emission tomography/computed tomography imaging for the diagnosis of immune checkpoint inhibitor-associated myocarditis. Arch. Cardiovasc. Dis..

[51] Ederhy S., Fenioux C., Cholet C., Rouvier P., Redheuil A., Cohen A., Salem J.-E. (2021). Immune Checkpoint Inhibitor Myocarditis With Normal Cardiac Magnetic Resonance Imaging: Importance of Cardiac Biopsy and Early Diagnosis. Can. J. Cardiol..

[52] Ida M., Nakamori S., Ishida M., Dohi K. (2021). Management of immune checkpoint inhibitor myocarditis: A serial cardiovascular magnetic resonance T2 mapping approach. Eur. Heart J..

[53] Sato T., Nakamori S., Watanabe S., Nishikawa K., Inoue T., Imanaka-Yoshida K., Ishida M., Sakuma H., Ito M., Dohi K. (2020). Monitoring of the Evolution of Immune Checkpoint Inhibitor Myocarditis with Cardiovascular Magnetic Resonance. Circ. Cardiovasc. Imaging.

[54] Haslbauer J.D., Lindner S., Valbuena-Lopez S., Zainal H., Zhou H., D’Angelo T., Pathan F., Arendt C.A., Bug G., Serve H. (2019). CMR imaging biosignature of cardiac involvement due to cancer-related treatment by T1 and T2 mapping. Int. J. Cardiol..

[55] Peleg Hasson S., Salwen B., Sivan A., Shamai S., Geva R., Merimsky O., Raphael A., Shmilovich H., Moshkovits Y., Kapusta L. (2020). Re-introducing immunotherapy in patients surviving immune checkpoint inhibitors-mediated myocarditis. Clin. Res. Cardiol..

